# Ab Initio investigation for DNA nucleotide bases sequencing using chiral carbon nanobelts and nanotubes

**DOI:** 10.1038/s41598-023-45361-3

**Published:** 2023-10-23

**Authors:** Seyyed Mostafa Monavari, Nafiseh Memarian

**Affiliations:** Faculty of Physics, Semnan University, P.O. Box: 35195-363, Semnan, Iran

**Keywords:** DNA nanotechnology, Nanoscale materials

## Abstract

Understanding the interaction mechanism between DNA nucleotide bases and carbon nanomaterials is an important issue in the field of identifying nucleotide molecules sequencing. In this article, the adsorption behavior of DNA nucleotide bases on the external surface of chiral carbon nanobelts (CNBs) (6, 5), (7, 6) and (8, 6), was comprehensively investigated from electronic and optical perspectives. As a result, it was determined that the DNA nucleotide bases have optical absorption in the ultraviolet region. When bases are adsorbed on the surface of CNBs, the optical absorption peak of the new complex structure shifted to the visible region. The study of the optical properties of selected CNBs showed that CNB (6,5) performs better in detecting Cytosine and the red shift in the absorption spectrum of complex structure is noticeable. Also, the effect of infinite length for chiral CNTs in DNA nucleotide base sequencing was investigated using DFTB approach. Our investigations based on electronic properties showed that CNTs have better performance than CNBs in DNA nucleotide base sequencing.

## Introduction

Genetic is a term used to denote something that pertains to genes or heredity. After identifying deoxyribonucleic acid (DNA) as the carrier of human genetic codes, researchers sought to determine the sequencing of the human genome and the Genome Project was the biggest human genes identification project^1^. Molecular analysis of DNA can diagnose over 400 diseases^2^. Knowing the gene sequence makes it possible to detect mutations in cells and the causes of emerging diseases. So that it is possible to prevent the transmitting of some genetic diseases to the next generations^3–5^. The nucleotide bases are nitrogenous biological compounds which are the building blocks of DNA and Ribonucleic acid (RNA). The DNA molecule consists of four nucleotide bases named as adenine (A), guanine (G), cytosine(C), and thymine (T). Where A and G are members of the purine group, and C and T belong to the pyrimidine group.

Therefore, detection of biological molecules (specially DNA nucleotide bases) is critical for health monitoring. This would lead to reduced medical costs and mortality globally. For example, it has been shown that early diagnosis of some diseases such as cancer increases the patient's chance of survival^2,6,7^. The development of electrochemical biological sensors depends on their integration with nanomaterials that increase their functionality and sensitivity.

Basically, in biological sensors, nanomaterials can be used as identifying agents for enzymes, antibodies, DNA, RNA and other proteins through their interaction or electron affinity for these biomolecules. Based on the interactions, the sensors are classified as follows: if the biomolecules are converted or degraded on the sensor surface, they are called catalytic sensors, and if they only interact with the sensor surface, they are classified as interaction-based biological sensors^8–10^.

Recently, much attention has been paid to the adsorption of biological molecules such as proteins, DNA nucleotide bases on the surface of nanostructures^11–14^ due to their potential therapeutic applications^15^. For example, examining the interactions of DNA nucleotide bases has become one of the important applications of carbon nanostructures in the DNA sequencing process^16–21^.

Carbon nanostructures such as graphene and CNTs are important components of modern biosensors due to their unique absorption, electrical and conductivity properties, as well as high mechanical strength and flexibility^22^. Chirality, which is defined by the chiral vector (n, m), determines the structural and electronic properties of carbon nanotubes (CNTs). Metallic and semiconducting CNTs can be classified based on $n-m=3l$ law (If $n-m=3l$, it is a metal, otherwise it behaves as a semiconductor^23^), resulting in different optical responses^24,25^.

Recent advances in the development of using CNTs have shown great potential in drug delivery systems, biological sensors and their proper implantation for the detection of viruses and especially the COVID-19 virus^26,27^, cancer cells, glucose, DNA, drug release carriers, volatile organic compounds and different inorganic gases. This is due to the high sensitivity of CNTs, which has made them the best choice for a biological sensor element^22^.

In this regard, extensive calculations and experiments have been performed to estimate the binding energy between DNA nucleotide bases and nanostructures. Due to their small size, carbon nanostructures allow us to also investigate interactions that occur with single molecules. The binding energy sequence of DNA nucleotide bases with carbon nanomaterials often follows the G > A > T > C order^28–34^. Theoretical calculations of CNT (5, 5) and CNT (10, 0) in gas phase have shown a similar sequence, but in aqueous solution the result A > G > T > C has been reported for the binding energy sequence^35^. In a report by Zhang et al., the adsorption of DNA nucleotide bases on different substrates such as CNTs, graphene, and C_60_ was investigated using DFT method. The results showed that guanine has the highest adsorption on the surface of CNT (6, 6), among all the nucleotide bases^36^. It has also been reported that the binding energy increases dramatically with increasing chirality, so that CNT (3, 3) < CNT (4, 4) < CNT (5, 5) and among the bases the binding energy is G > T ∼ A > C. It is also stated that the changes in HOMO and LUMO were not very noticeable and therefore no gap difference occurred^37^. In a study by Umadevi et al.^37^, the sequencing of DNA nucleotide bases with graphene and CNTs based on density functional theory with the Mo6-2X functional was investigated. They concluded that the binding energy of the CNTs with DNA nucleotide bases follows the order G > T ≈ A > C, and the interaction of graphene with these DNA nucleotide bases follows the order G > A > T > C. Eslami et al.^38^, based on DFT, obtained the magnitude of the interaction energy of boron–carbon graphene nanosheets with DNA nucleotide bases as A > T > C > G in solvent-free conditions and C > T > G > A in the presence of solvent.

Numerous experimental studies have also obtained different binding energies for DNA nucleotide bases interacting with CNTs and graphitic crystals, due to the different experimental conditions^39,40^. For instance, Li et al.^41^, by creating nanopores made of single-walled CNTs on a silicon wafer and measuring the change in the ionic current passing through the nanotubes in the presence and absence of DNA molecule, were able to perform sequencing. they produced CNTs with diameters of 1 to 2 nm. Also, another experimental research was performed using CNTs (10, 10) with the diameter of 2 nm, which also reported a change in the ionic current passing through the nanotube due to the presence of DNA molecule^42^. CNBs have similar functional properties to CNTs, but limited length, so their electronic properties depend on their length. Therefore, the study of CNBs in the properties of biosensors is important. Proper differentiation of nucleotide bases binding energies is a major challenge for experimental and theoretical research. Therefore, despite the work done so far, there is a need for extensive theoretical research in the field of DNA nucleotide bases sequencing.

In this article, we investigate the interaction of DNA nucleotide bases with chiral CNBs (6, 5), (7, 6), and (8, 6) using DFT method. The sequencing degree between DNA nucleotide bases by CNBs is examined by studying the intensity of van der Waals bonding and then by studying optical properties. Our results showed that among the three considered chiral CNBs, CNB (6, 5) performs better than other CNBs in the DNA nucleotide bases sequencing. In the next step, to find out the bases sequencing by chiral CNTs, we have studied the electronic properties of DNA nucleotide bases adsorbed on the outer surface of CNT (6, 5) and CNT (7, 6). Since Chiral CNT (6, 5) and (7, 6) were experimentally synthesized^43^, they have been chosen for this study.

## Simulation method

The most important factor considered in this study is the sequencing of the bases' binding energies by the CNBs and CNTs. The B3LYP hybrid functional^44^, with considering the van der Waals interaction, includes the Becke three-parameter exchange term and contains the LYP correlation functional. Despite its high computational cost, this functional has higher accuracy in determining bond energy compared to other functionals by reducing the self-energy error, but like other common functionals, it predicts the hydrogen bond binding energy less than the actual value. To solve this problem, DFT-D3 van der Waals correction was used to increase the accuracy of calculations.^45^. We applied B3LYP functional and imported atomic orbitals using the LANL2DZ basis set was selected for expanding the system's wave function^46^. All structures were initially optimized to a minimum energy structure, such that the interatomic force reached less than 0.02 eV/Å. The Absorption spectra were calculated in the TD-DFT framework. All the calculations of CNBs were performed using the Gaussian 98 package^47^. The electron density of states (DOS) and Absorption spectra were obtained using GaussSum software^48^.

Band structure calculations for CNTs (6, 5), (7, 6) and nucleobase molecules near CNTs were done based on Slater–Koster Tight-Binding Model (DFTB) as implemented in the DFTB + package^49^, using 1 × 1 × 50 BZ k-point. To solve the Kohen-Sham equations, semi-empirical Slater-Koster CHNO basis sets have been used with the generalized gradient approximation (GGA)^50^.

**Figure 1:**
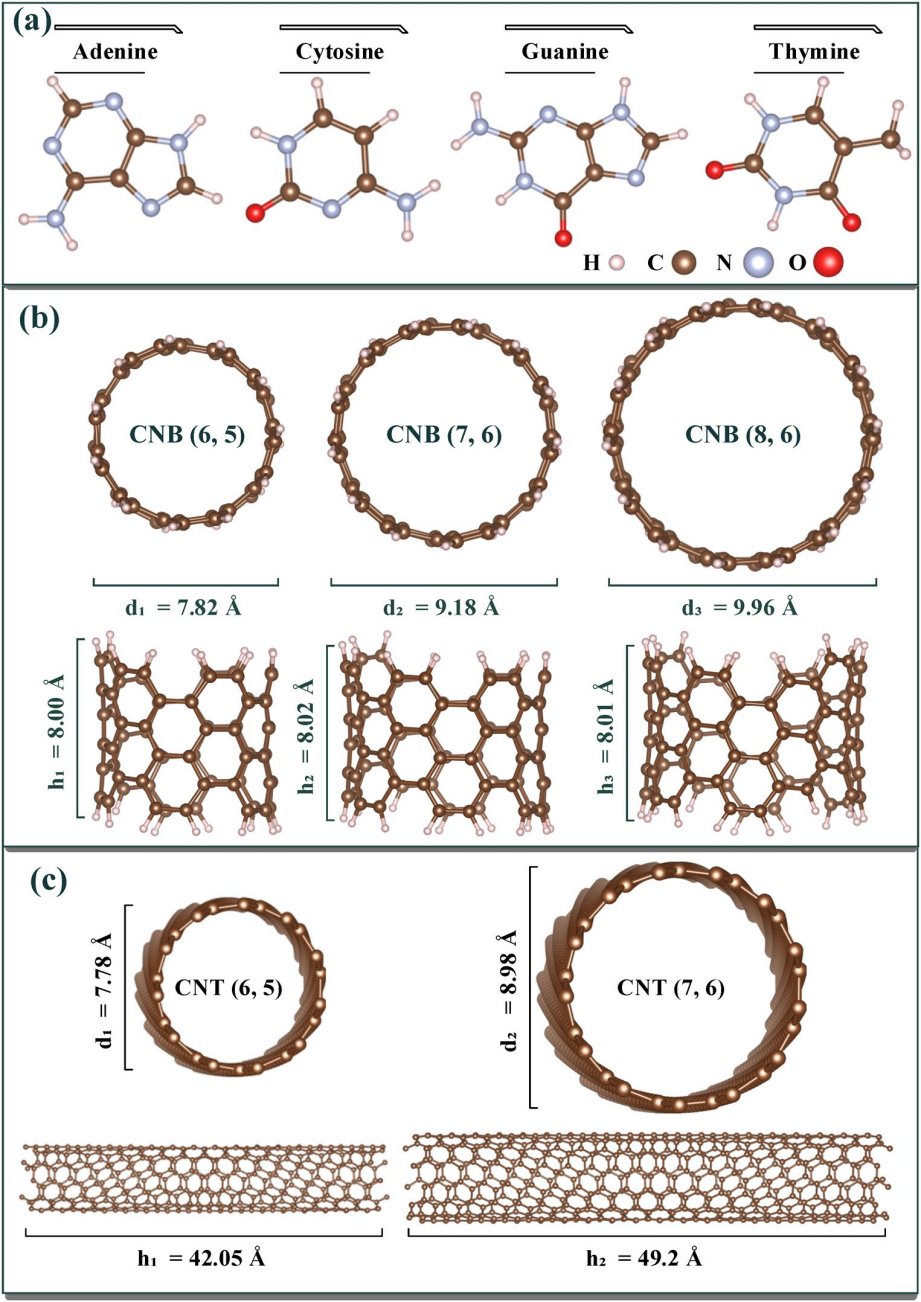
Optimized structures of (**a**) DNA nucleotide bases; Cross-sectional and side view of (**b**) CNB (6, 5), (7, 6), (8, 6); and (**c**) CNT (6, 5), (7, 6).

Calculations of electronic properties were performed at room temperature (300 K). Both the supercell volume ranges and the position of the atoms inside the supercell have conditions that the tolerance of force and energy convergence is less than 0.05 kcal/mol/Å and 0.01 kcal/mol, respectively. To optimize the conjugate gradient algorithm by applying periodic boundary conditions (PBC) along the growth orientation, it has been used for all structures. The optimized structures of DNA nucleotide bases, CNBs and CNTs with the above-mentioned methods are shown in Fig. 1a–c, respectively.

## Results and discussion

### Sequencing of DNA nucleotide bases by chiral CNBs

The binding energy was calculated from the difference between the total energy of the complex structures (nucleotide bases + CNBs) and the sum of the energies of the isolated CNBs and Nucleotide bases ($\Delta {E}_{B}= {E}_{CNBs+DNA bases}- {E}_{CNBs}- {E}_{DNA bases}$).

In the first step, we are looking for parameters to be able to make a sufficient distinction between these four Nucleotide bases. Electrochemical properties are factors that can be used to sequence materials. These properties include highest occupied molecular orbital (HOMO) and the lowest unoccupied molecular orbital (LUMO) energy, H–L energy gap (${E}_{gap}= {E}_{LUMO}- {E}_{HOMO}$), the variation in the band gaps in the presence of the DNA nucleotide bases with respect to the pristine CNTs (${\Delta E}_{gap}= {E}_{gap}\left(CNT\right)- {E}_{gap}\left(CNT+DNA bases\right)$), chemical potential ($\mu = \frac{{E}_{HOMO} + {E}_{LUMO}}{2}$), ionization potential ($IP= - {E}_{HOMO}$), electron affinity ($EA= - {E}_{LUMO}$), chemical hardness ($\eta = \frac{{E}_{LUMO} - {E}_{HOMO}}{2}$), electrophilicity ($\omega = \frac{{\mu }^{2}}{2\eta }$), binding energy $(\Delta {E}_{B})$, and dipole moment vector (D) was shown in Table 1.

**Table 1:** Electrochemical properties for DNA nucleotide bases, CNBs and complex structures.

Structure	HOMO	LUMO	E_gap_	ΔE_gap_	Μ	IP	EA	η	ω	ΔE_B_	D
Adenine	− 6.33	− 1.07	5.26	–	3.70	6.33	1.07	2.63	2.60	–	2.62
Cytosine	− 6.48	− 1.35	5.13	–	3.92	6.48	1.35	2.56	2.99	–	7.41
Guanine	− 6.01	− 0.66	5.35	–	3.33	6.01	0.66	2.67	2.08	–	7.46
Thymine	− 6.93	− 1.64	5.29	–	4.29	6.93	1.64	2.64	3.48	–	4.90
CNB (6, 5)	− 4.94	− 2.57	2.38	–	3.75	4.94	2.57	1.19	5.93	–	0.12
CNB (6, 5) + Adenine	− 4.95	− 2.58	2.37	0.01	3.77	4.95	2.58	1.19	5.99	0.25	2.04
CNB (6, 5) + Cytosine	− 5.17	− 2.80	2.37	0.01	3.99	5.17	2.80	1.19	6.71	0.13	6.22
CNB (6, 5) + Guanine	− 5.11	− 2.75	2.36	0.02	3.93	5.11	2.75	1.18	6.54	0.16	7. 21
CNB (6, 5) + Thymine	− 5.00	− 2.62	2.37	0.01	3.81	5.00	2.62	1.19	6.12	0.26	4.26
CNB (7, 6)	− 4.89	− 2.62	2.26	–	3.76	4.89	2.62	1.13	6.24	–	0.13
CNB (7, 6) + Adenine	− 4.91	− 2.65	2.25	0.01	3.78	4.91	2.65	1.13	6.34	0.23	1.93
CNB (7, 6) + Cytosine	− 4.65	− 2.40	2.25	0.01	3.96	4.65	2.40	1.13	5.52	0.31	9.80
CNB (7, 6) + Guanine	− 4.98	− 2.72	2.26	0	3.85	4.98	2.72	1.13	6.57	0.16	7.71
CNB (7, 6) + Thymine	− 4.95	− 2.69	2.26	0	3.82	4.95	2.69	1.13	6.45	0.26	4.35
CNB (8, 6)	− 4.55	− 2.96	1.59	–	3.76	4.55	2.96	0.80	8.86	–	0
CNB (8, 6) + Adenine	− 4.57	− 2.97	1.59	0	3.77	4.57	2.97	0.80	8.91	0.25	1.93
CNB (8, 6) + Cytosine	− 4.76	− 3.17	1.59	0	3.97	4.76	3.17	0.79	9.91	0.13	10.11
CNB (8, 6) + Guanine	− 4.66	− 3.06	1.59	0	3.86	4.66	3.06	0.80	9.35	0.17	7.76
CNB (8, 6) + Thymine	− 4.61	− 3.01	1.59	0	3.81	4.61	3.01	0.80	9.12	0.27	4.36

The most important factor considered in this section is the differentiation of the DNA nucleotide bases' binding energies by the sensor substrate (CNBs and CNTs) from each other. According to the calculations, most of the complex structures in Table 1 have weak hydrogen bonding (less than 0.2 eV^51^). Only some samples such as guanine + CNB (6, 6) have a very small difference in the medium bond energy range (between 0.2 eV and 0.65 eV^51^) and benefit from a suitable hydrogen bond strength.

The values of the dipole moment vector (D) for the isolated system and in the complex mode are shown in Table1. From Fig. 1 (b), the symmetry order in the CNB structures is CNB (8, 6) > CNB (6, 5) > CNB (7, 6), so it can be said that in CNB (8, 6), due to the higher spatial symmetry, the dipole moment is ${3.8\times 10}^{-5}$ Debye, which is practically considered zero in Table 1. The electric dipole moments in DNA nucleotide bases are in the order of G > C > T > A. Guanine and cytosine, has more electronegative atoms (such Oxygen) and consequently higher dipole moment. The dipole moment of our complex structures is also investigated. For the CNB (6, 5) + DNA nucleotide bases system, the dipole moment was obtained as CNB + G > CNB + C > CNB + T > CNB + A, which showed the same order of dipole moment for the pure nucleotide bases. For CNBs (7, 6) and (8, 6), the dipole moment was as CNB + C > CNB + G > CNB + T > CNB + A. It can be said that guanine in CNB (6, 5) + nucleotide bases and cytosine in CNBs (7, 6) and (8, 6) + nucleotide bases have the highest dipole moment. Therefore, it can be concluded that as the DNA nucleotide bases get closer to the CNBs, the uniform charge distribution of the CNBs has been changed due to the nonzero dipole moment of bases.

**Figure 2:**
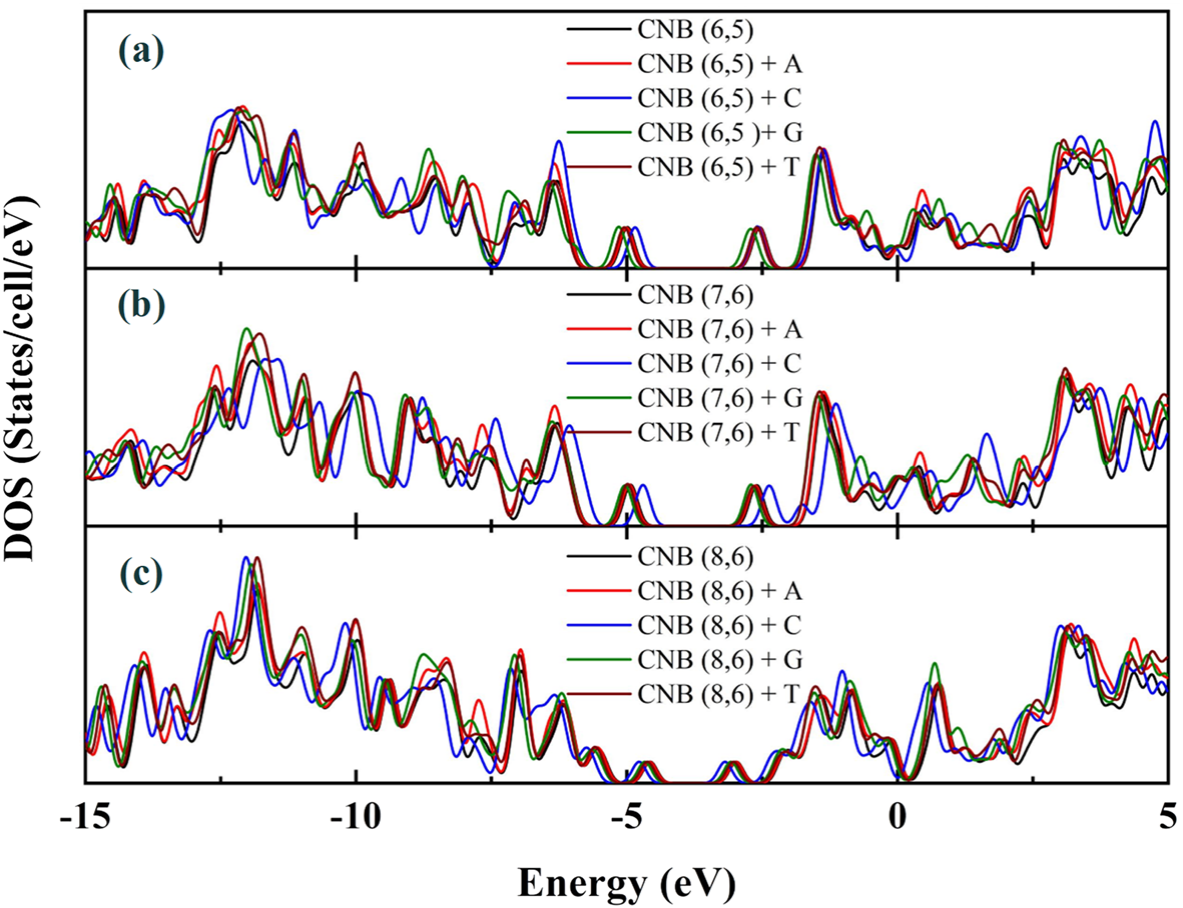
DOS of (**a**): pristine CNB (6, 5) with CNB (6, 5) + DNA nucleotide bases complex structures, (**b**): pristine CNB (7, 6) with CNB (7, 6) + DNA nucleotide bases complex structures, (**c**): pristine CNB (8, 6) with CNB (8, 6) + DNA nucleotide bases complex structures.

The electron felicity (ω) shows the relative ability of an atom to attract a pair of bonding electrons to itself and describes the nature of the interaction between two molecules. Here, the presence of electronegative oxygen and nitrogen atoms in DNA nucleotide bases caused the high electron attraction of cytosine and guanine nucleobases to the carbon atoms of CNBs. Chemical hardness is defined as the resistance to the change of electron distribution^52^. The results showed that the addition of DNA nucleotide bases to CNBs does not show many changes in chemical hardness, and the DOS spectrum does not change. This issue is also in agreement with Fig. 2. As shown in Table 1, due to the presence of oxygen and nitrogen atoms in DNA nucleotide bases, the chemical potential of complex structure has increased compared to the pure CNBs. That is, the electron can be more easily excited and lead to the ionization of nanostructures, with less required energy. Also, the results of chemical potential showed that the highest chemical potential belonged to CNB (7, 6) + C and the lowest belonged to CNB (6, 5) + C.

The results of electron density of state (DOS) calculations are presented in Fig. 2 showed that the pairs of non-bonded electrons in the oxygen and nitrogen atoms of DNA nucleotide bases could not have serious effect on the HOMO levels and did not impose new energy states between the energy gap region, so the DOS do not have a severe change.

**Figure 3:**
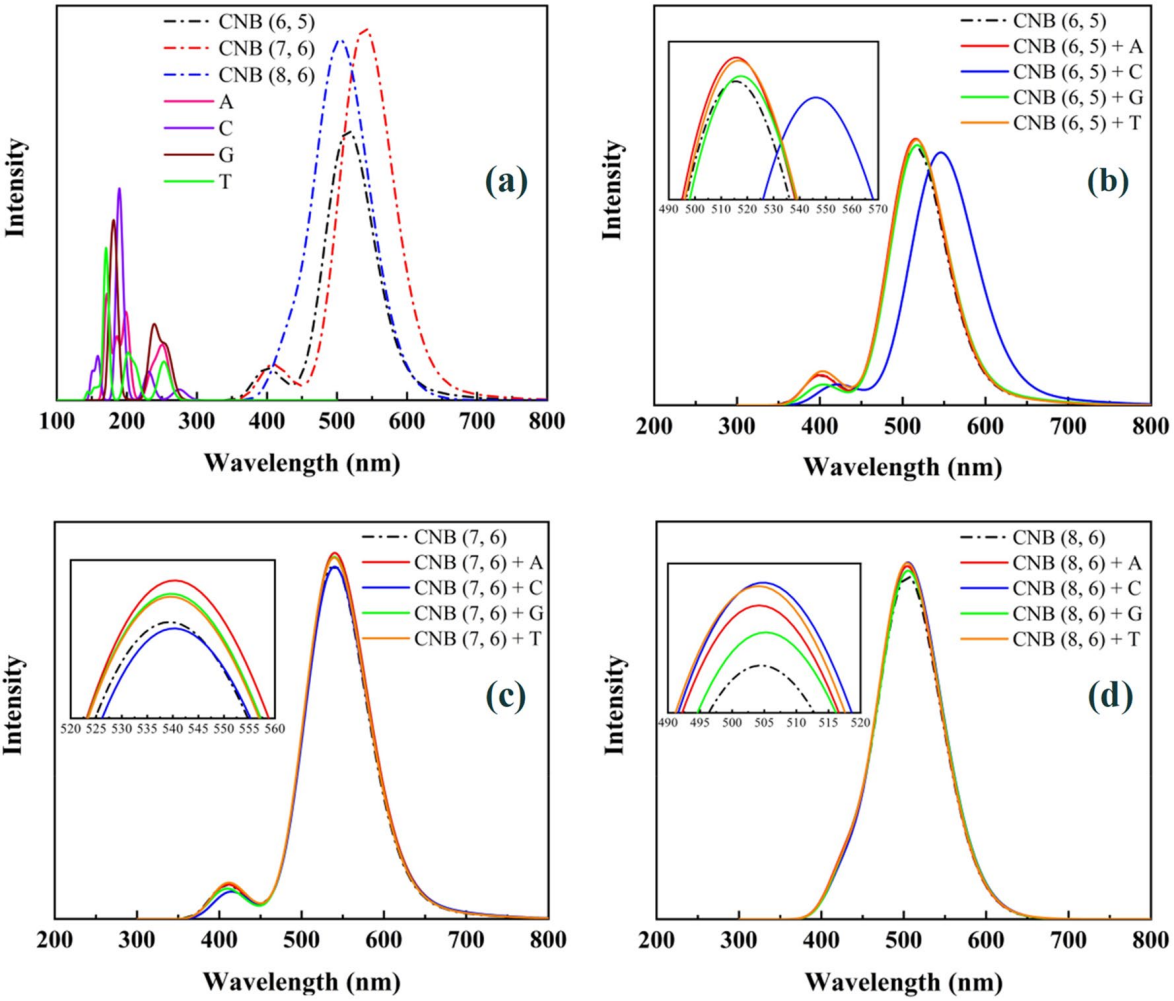
TD-B3LYP predicted absorption spectrum of (**a**) pristine CNB, DNA nucleotide bases molecules, and (**b**–**d**) CNBs + DNA nucleotide bases complex.

Furthermore, we have calculated the optical properties of CNBs to sequence the DNA nucleotide bases. In the following, we show how the absorption spectrum of CNB changes with the presence of DNA nucleotide bases. The predicted spectra of CNBs and CNBs + nucleotide bases complex structures are shown in Fig. 3. The results also show that the absorption of DNA nucleotide bases is in the ultraviolet region and there is almost no absorption in the visible region for them. What causes the distinction in the absorption spectrum of different chirality's is: First, the remarkable difference in their absorption wavelengths, and second: the difference in absorption intensity, both of which cause the difference in the resulting color. As this shows the high potential of this sensing material in detecting Nucleotide bases. So that among the CNBs, CNB (7, 6) with a wavelength of 539 nm is in the light green wavelength range. CNB (8, 6) and CNB (6, 5) with wavelengths of about 504 nm and about 515 nm are in the dark green wavelength range. Among these, only CNB (6, 5) + C with a 40 nm red shift relative to CNB (6, 5) is in the yellow region. So. CNB (6, 5) could act as a biosensor of Cytosine nucleotide base.

**Figure 4:**
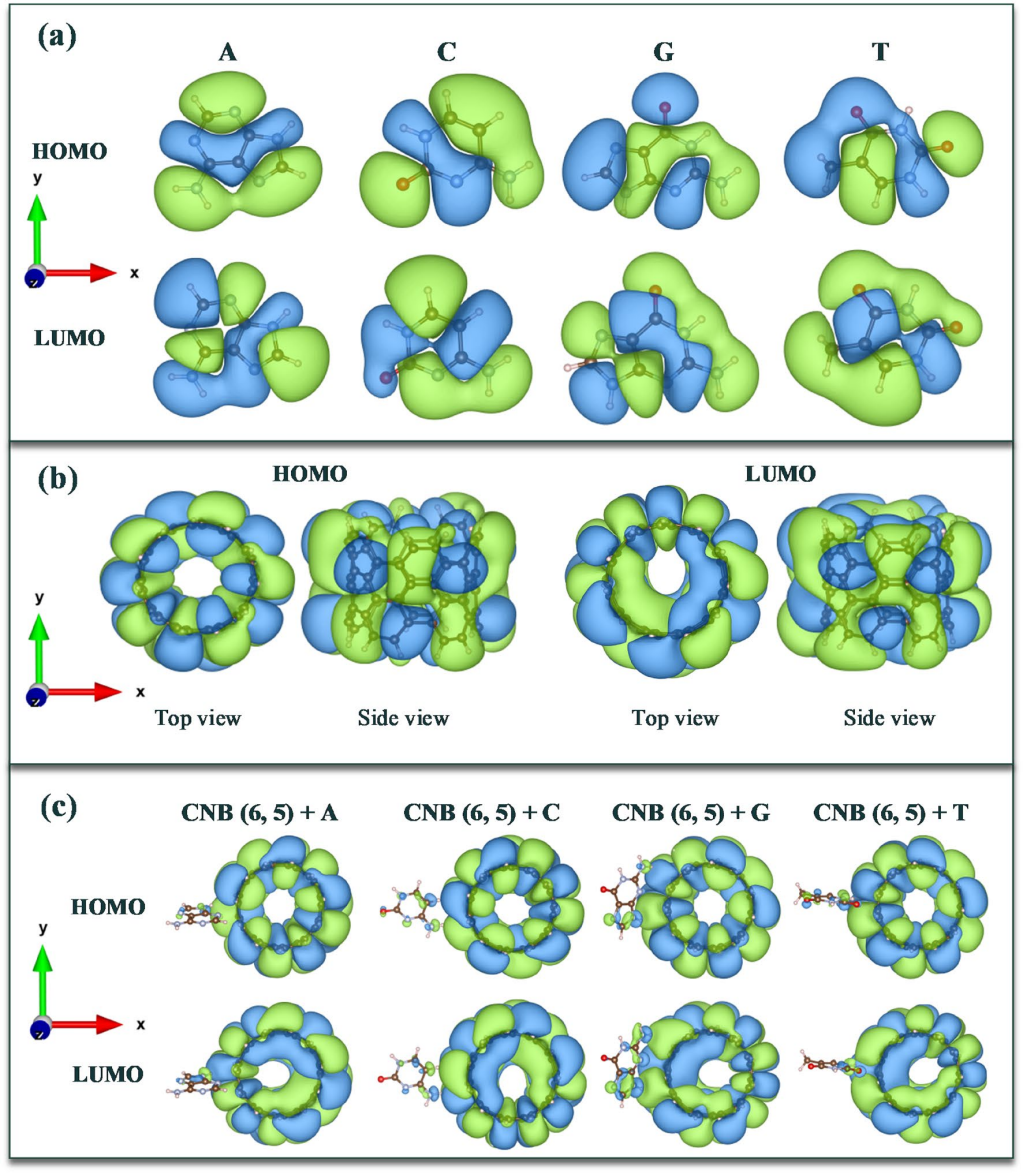
HOMO and LUMO isosurfaces for (**a**) Nucleotide bases, (**b**) pristine chiral CNB (6, 5), and (**c**): CNB (6, 5) + nucleotide bases.

HOMO, and LUMO isosurfaces are very important in investigating electronic properties, because by studying HOMO and LUMO energy and the difference between these two parameters, i.e., the H–L energy gap, important information can be obtained. For this purpose, we have examined the electronic changes in the CNBs with nucleotide bases complex using HOMO, and LUMO isosurfaces. As shown in Fig. 4a–c, HOMO and LUMO isosurfaces are plotted for DNA nucleotide bases, CNBs and CNBs + DNA nucleotide bases complex structure, respectively. It should be noted that, HOMO and LUMO isosurface are plotted with isovalue = 0.002 e/Å^3^.

In all the studied systems, the HOMO energy level of DNA nucleotide bases increases and their LUMO energy level decreases. As a result of the reduction in the LUMO level, the load arrangement has been changed. These charge distribution changes occurred due to the formation of hydrogen bonds between DNA nucleotide bases and nanobelts, such that bases were electron donors and CNBs were electron acceptors, and charge transfer from DNA nucleotide bases to CNBs was carried out. The results showed that guanine nucleobase has caused the highest change in the HOMO and LUMO distribution of CNB (6, 5) by electric charge transfer of from guanine nucleobase to CNB (6, 5). This has made CNB (6, 5) a good candidate as the guanine sensor.

### Sequencing of DNA nucleotide bases by chiral CNTs

In this research, to sequence DNA nucleotide bases by CNTs, we sought to select nanotubes that are expected to be non-metallic, as well as they could be made experimentally and showed suitable electronic properties. Therefore, two types of chiral carbon nanotubes, CNT (6, 5) and CNT (7, 6) were selected. As expected, the results showed that chiral CNT (6, 5) and CNT (7, 6) are semiconductors. This is consistent with the results of other researchers^27,53^. To obtain electronic properties for structures with high number of atoms (many body systems), using the DFTB can be very useful. Since the number of atoms in the unit cell of selected CNTs are more than 150 atoms (Table 2) and the unit cell can be repeated in the direction of nanotube growth, the calculations in DFTB approach was performed.

**Table 2:** Number of atoms in the simulation super cell for CNTs + Nucleotide bases, the electronic band gaps (${E}_{gap}$), and the variation in band gap ($\Delta {E}_{gap}$), compared to pristine CNTs.

Structure	Number of atoms in the unit cell	E_gap_ (eV)	ΔE_gap_ (eV)
CNT (6, 5)	364	0.934–(0.967^27^, 1.272(Exp.)^53^)	–
CNT (7, 6)	508	0.789–(0.821^27^–, 1.105(Exp.)^53^)	–
CNT (6, 5) + Adenine	379	0.780	0.154
CNT (6, 5) + Cytosine	377	0.496	0.438
CNT (6, 5) + Guanine	380	0.416	0.518
CNT (6, 5) + Thymine	379	0.933	0.001
CNT (7, 6) + Adenine	523	0.695	0.094
CNT (7, 6) + Cytosine	521	0.405	0.384
CNT (7, 6) + Guanine	524	0.309	0.48
CNT (7, 6) + Thymine	523	0.789	0

The calculation results of the electronic properties including the energy gap (${E}_{gap}$) and the energy gap difference ($\Delta {E}_{gap}$) between CNTs + DNA nucleotide bases complex and pristine CNT structures for CNT (6, 5) and CNT (7, 6) are shown in Table 2. The results showed that the addition of DNA nucleotide bases molecules causes significant changes in the energy gap of carbon nanotubes, so that for CNT (6, 5) the average of these changes is 0.28 eV and for CNT (7, 6) is 0.24 eV. In other words, among these two nanotubes, CNT (6, 5) shows a better behavior in DNA base sequencing and can be a suitable candidate for DNA bases diagnostic biosensor.

**Figure 5:**
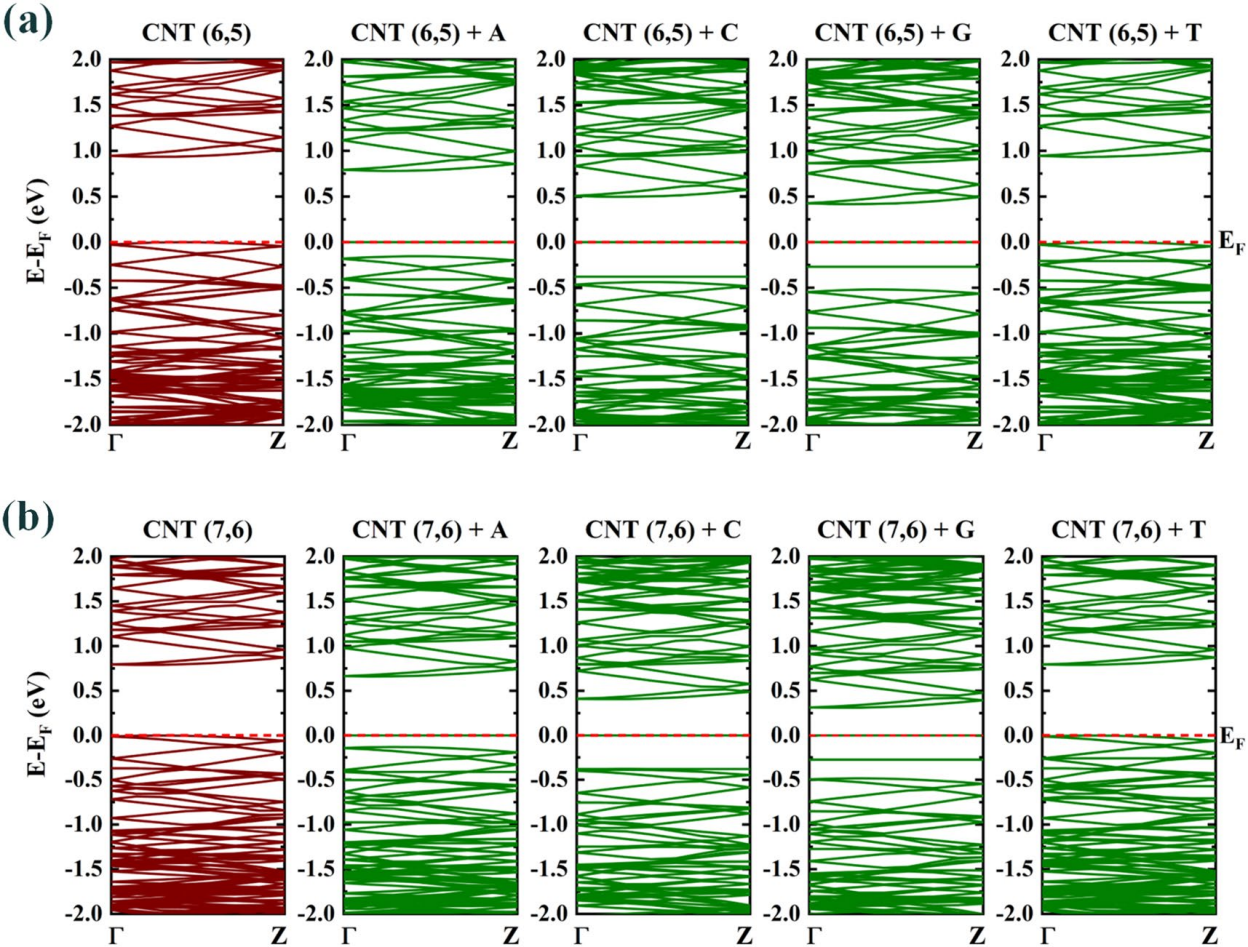
Top: (**a**) the corresponding DFTB + electronic band structures for chiral CNT (6, 5) + bases complex structure. Down: (**b**) the corresponding DFTB + electronic band structures for chiral CNT (7, 6) + DNA nucleotide bases complex structure. Fermi energy (EF) is set to zero.

The addition of guanine molecule to the environment of CNT (6, 5) causes the greatest reduction of its energy gap from 0.934 eV to 0.416 eV. Therefore, it can be said that the gap changes in the amount of 0.518 eV happened here. This behavior can also be seen for CNT (7, 6) and among Nucleotide bases, guanine has the largest energy gap change, which is 0.48 eV. Also, the order of energy gap changes when the DNA nucleotide bases approach the external environment of CNT (6, 5) and CNT (7, 6) have a similar behavior and are CNTs + G > CNTs + C > CNTs + A > CNTs + T. Figure 5 shows the energy band structure of CNT (6,5) and CNT (7,6) in brown color. In addition, Fig. 5 shows the effect of entering DNA nucleotide bases in the band structure of CNTs in green color. It should be mentioned that the Fermi energy was set to zero and the space k is drawn in the first Brillouin zone from the path Γ to Z.

**Figure 6:**
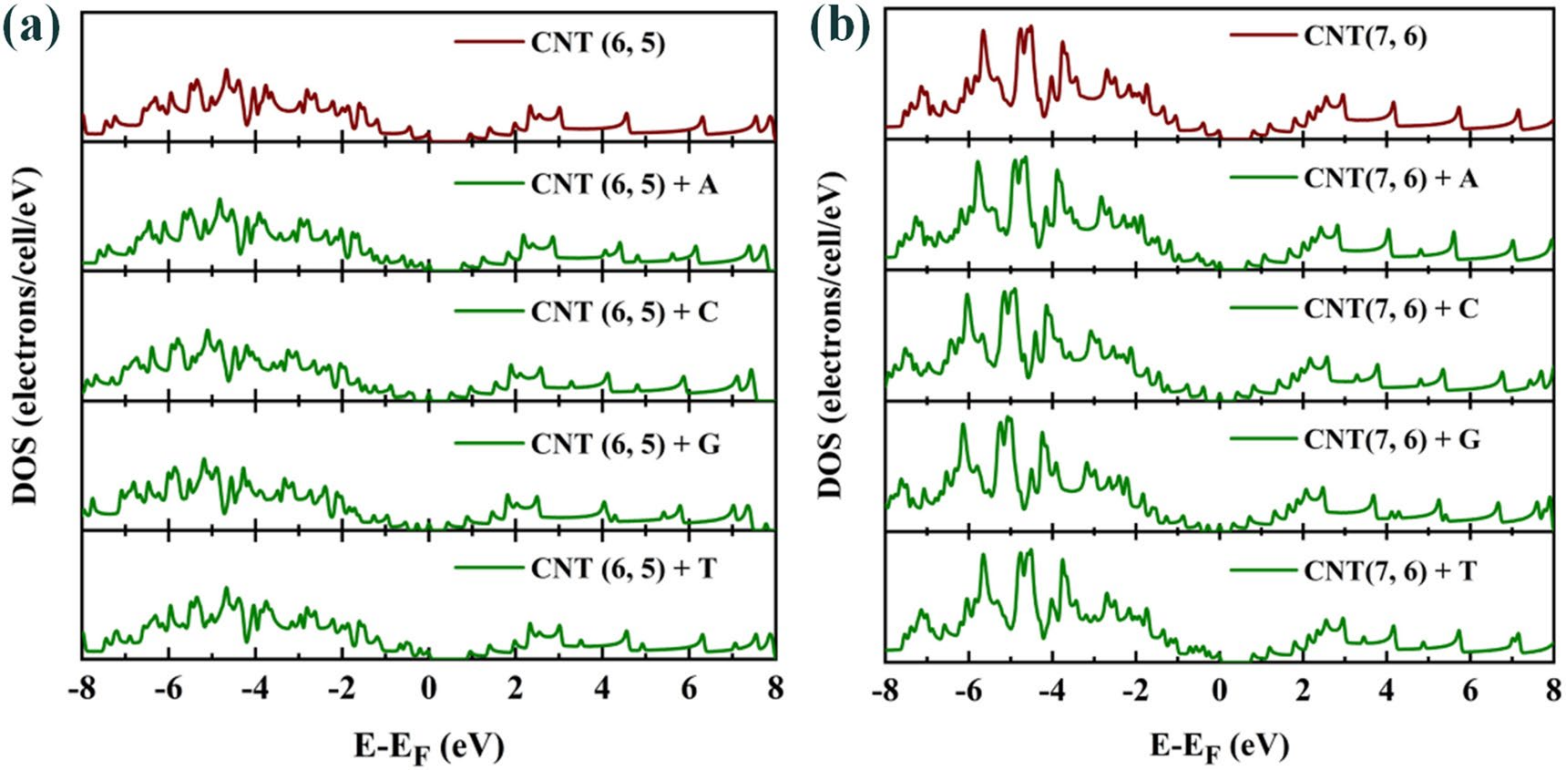
DOS spectrum of (**a**): pristine CNT (6, 5) and CNT (6, 5) + DNA nucleotide bases complex structure; (**b**): pristine CNT (7, 6) and CNT (7, 6) + DNA nucleotide bases complex structure.

In the following, the electron DOS spectra for CNTs and CNTs + DNA nucleotide bases complex structures are presented in Fig. 6. The results indicate that a slight increase in density of states due to the increase in the number of atoms in the unit cell. As mentioned before and in Fig. 5, the effect of guanine presence on chiral CNTs is more evident than other nucleotide bases. In fact, it can be said that these nucleotides have reduced the band gap of selected CNTs by creating inter-gap states.

## Conclusion

In this paper, the feasibility of using carbon nanobelts and nanotubes for the DNA nucleotide bases sequencing have been studied. The study was conducted based on DFT and DFTB methods. The results showed binding energy depend on the chirality of CNB (6, 5) and CNB (8, 6) as C > G > A > T, while for the CNB (7, 6), binding energy is as G > A > T > C. The binding energy results indicate physical adsorption between DNA nucleotide bases and selected CNBs by non-covalent bonding. By examining HOMO, and LUMO isosurfaces, it was found that charge transfer from DNA nucleotide bases to CNBs occurred. The guanine nucleobase showed the most charge transfer to CNB (6, 5). Optical absorption spectra of CNB (6, 5) had a better performance in sequencing DNA nucleotide bases than the others and was especially excellent in cytosine base detection.

we also studied semiconducting CNT (6, 5) and CNT (7, 6), which have been synthesized experimentally, in sequencing nucleotide bases. We observed that CNTs perform better than CNBs in detecting and distinguishing DNA nucleotide bases and show more energy gap changes. Among nucleotide bases, guanine showed the most energy gap changes when it enters the external environment of CNT (6, 5) and CNT (7, 6). Therefore, CNT (6, 5) and CNT (7, 6) are suitable candidates for making a biosensor substrate for DNA nucleotide bases diagnosis, especially guanine. This paper shows the ability of selected CNBs and CNTs in separation and sequencing of Nucleotide bases.

## Data Availability

The datasets used and/or analyzed during the current study are available from the corresponding author upon reasonable request.
